# Inflammatory Bowel Diseases: Is There a Role for Nutritional Suggestions?

**DOI:** 10.3390/nu13041387

**Published:** 2021-04-20

**Authors:** Lorenzo Bertani, Davide Giuseppe Ribaldone, Massimo Bellini, Maria Gloria Mumolo, Francesco Costa

**Affiliations:** 1 Department of Translational Research and New Technologies in Medicine and Surgery, University of Pisa, 56100 Pisa, Italy; lorenzobertani@gmail.com (L.B.); mbellini58@gmail.com (M.B.); 2 Department of Medical Sciences, University of Turin, 10126 Turin, Italy; davidegiuseppe.ribaldone@unito.it; 3 IBD Unit, Department of General Surgery and Gastroenterology, Pisa University Hospital, 56124 Pisa, Italy; g.mumolo@int.med.unipi.it

**Keywords:** IBD, nutrition, micronutrients, macronutrients, Crohn’s disease, ulcerative colitis, anemia, sarcopenia

## Abstract

Nutrition has an important impact on inflammatory bowel diseases (IBD). In particular, several studies have addressed its role in their pathogenesis, showing how the incidence of IBD significantly increased in recent years. Meanwhile, nutrition should be considered a component of the treatment of the disease, both as a therapy itself, and especially in the perspective of correcting the various nutritional deficiencies shown by these patients. In this perspective, nutritional suggestions are very important even in the most severe forms of IBD, requiring hospitalization or surgical treatment. Although current knowledge about nutrition in IBD is increasing over time, nutritional suggestions are often underestimated by clinicians. This narrative review is an update summary of current knowledge on nutritional suggestions in IBD, in order to address the impact of nutrition on pathogenesis, micro- and macro-nutrients deficiencies (especially in the case of sarcopenia and obesity), as well as in hospitalized patients.

## 1. Introduction

Inflammatory bowel diseases (IBD) are chronic relapsing diseases of unknown origin affecting the gastrointestinal tract [1]. Diet is one of the key factors in their pathogenesis and outcome, since it could have a pro-inflammatory effect [2]. Notably, the incidence of IBD worldwide has significantly increased in the last decades [3,4], and it could be related to differences in lifestyle, including the adoption of a western diet, characterized by high amounts of proteins and saturated fats, in concomitance with low amounts of vegetables, fibers, and fruits [5]. 

Patients with IBD are particularly aware of the dietary influence on their disease, since approximately 70% of them think that diet could influence their condition [6], 60% consider diet to play a major role in inducing a relapse [7], and 16% are convinced that diet could initiate the disease [8]. Interestingly, in order to modify their dietary habits, patients frequently tend to avoid certain foods rather than increasing the intake of dietary components with presumably more beneficial properties [9]. 

Several dietary factors have been suggested to have a potential causative role in IBD [10]. On the other hand, diet components may have therapeutic implications, being able to correct nutritional deficiencies as well as to exert anti-inflammatory properties [11].

However, although in recent years, clinical and experimental research significantly increased the therapeutic armamentarium [12], few data are currently available for dietary suggestions. Some guidelines included specific formula diets as induction or adjunctive therapy for Crohn’s disease (CD), while there is no specific recommendation for ulcerative colitis (UC) [13]. This narrative review aims to summarize current knowledge on nutritional suggestions in IBD, with the perspective to address the impact of nutrition on pathogenesis, micro- and macro-nutrients deficiencies (especially in the case of sarcopenia and obesity), as well as in hospitalized patients.

## 2. Nutrition in IBD Pathogenesis

IBD are multifactorial diseases in which genetic predisposition, altered immune system, dysbiosis, and environmental factors contribute to disease onset and recurrence [1].

In the last decades, the incidence of IBD in developing countries, but not in the Western world, increased significantly: Since genetic changes manifest over large time frames, external factors have been called into question [4]. Migrants from low- to high-prevalence regions tend to acquire a “high-prevalence” pattern of IBD onset in two generations [14]. The adoption of a low-fiber diet, rich in saturated fats, refined sugars, processed foods, associated with a diet-induced gut dysbiotic profile, appears to play a major role in the link between environment and inflammation [5,15]. 

### 2.1. Fat and IBD

#### 2.1.1. Saturated Fats

Saturated fats are found mainly in animal products, such as meat and dairy products.

In-vivo studies in animal models found that a diet rich in high saturated fats promotes chronic inflammation [16], although the mechanism is still unknown. One explanation is that the amino acid taurine, present in saturated fats, linked to bile acids, seems to increase substrate availability for sulfur-reducing bacteria like *Bilophila Wadsworthia*, highly prevalent in the dysbiotic microbiota of IBD patients. Furthermore, Muhomah et al. [17] found that saturated fats are able to reduce the level of secretory immunoglobulin (sIg) A, altering the immune response to intestinal microbiota. 

In addition to the risk of developing IBD, a diet rich in saturated fatty acids seems to increase the relapse risk, particularly in UC [18].

#### 2.1.2. Polyunsaturated Fats (Omega-3)

Omega-3 fats, contained in olive oil, are powerful antioxidants and are associated with a lower risk of IBD, both in UC [19,20], as well as CD: Children that consume a diet rich in omega-3 fats are at lower risk of CD development [21].

### 2.2. Red Meat and IBD

The consumption of red meat, due to its high content of saturated fat and cooking method, has been linked to an higher risk of colon cancer and inflammation [22]. Red meat is metabolized by intestinal bacteria with production of branched-chain amino acids and toxic elements like hydrogen sulfide, nitrous compounds, amines, and ammonia that induce DNA damage of eucaryotic cells and promote colon inflammation in murine models [23,24]. Notably, large epidemiological data confirmed the association between high consumption of red meat and risk of IBD development, in particular UC [25], where it was found to affect also the relapse risk [26].

### 2.3. Sugars and IBD

Sugars are part of everyone’s diet. The modern diets are rich in sugar; chocolate, cookies, cakes, ice creams, fruit punches, energy drinks, soft drinks, iced tea, and lemonade contribute to the “pandemic” of obesity, diabetes, and steatosis that plagues the western world [27]. Sucrose, a disaccharide composed of monosaccharides fructose and glucose, is the most common sugar found in processed foods. Many studies have linked high sugar intake with IBD incidence [28,29,30,31].

The pathogenic mechanism that links high sugar intake with the onset of IBD is not fully elucidated. A possible explanation is a reduction of intestinal mucus [32]. A sugar-rich diet favors the increase of *Akkermansia muciniphila*, a mucolytic bacterium. The mucus layer separates luminal bacteria from intestinal epithelium: A thinner mucus layer allows bacteria to come in contact with the epithelial cells, eliciting an inflammatory response. In addition, this type of diet increases the percentage of pro-inflammatory *Sutterellaceae* and *Marinilabiliaceae*, which induce bowel inflammation [33], and reduce bacteria with anti-inflammatory properties like *Lachnospiraceae* and *Lactobacillaceae*, able to produce the short-chain fatty acid (SCFA) butyrate, the main anergy source of enterocytes [34]. 

### 2.4. Fiber and IBD

In contrast to eastern and African diets, the western diet is low in fiber. Butyrate-producing bacteria, like those belonging to Bacteroidetes and Firmicutes phylum, exert their anti-inflammatory activity metabolizing dietary fibers: A low-fiber diet leads to reduced production of butyrate, which acts as a negative regulator of pro-inflammatory pathways and enhances the intestinal barrier function [35]; this, in turn, increased the risk of IBD onset [36]. When substrate is scarce, intestinal bacteria use the intestinal mucus as a nutrient, which leads to inflammation through close contact between bacteria and the epithelial layer [37]. Long-term intake of fibers from fruit has been shown to be protective against the development of CD, but not of UC [38,39,40,41,42]. 

The benefit of fibers in IBD remission is uncertain. Brotherton et al. [43] found that a low fiber consumption in CD during remission is associated with a higher risk of clinical relapse, but in the case of stricturing CD, the consumption of dietary fibers could precipitate obstruction. 

### 2.5. Nuts and IBD

A diet rich in nuts and dried fruits, due to their high content of omega-3 fatty acids, fibers, and antioxidants, has been shown to decrease the cardiometabolic and inflammatory risk; the beneficial effects on health include reduced risk of cancer, diabetes, neurological diseases [44]. A diet rich in walnuts protects mice from dextran sulfate sodium (DSS)-induced colitis [45]. The mechanism of action seems to be a decrease of macrophages activation, reduction in the production of pro-inflammatory cytokines like IL-6, IL-8, IL-1α, tumor necrosis factor (TNF), and an increase of anti-inflammatory cytokine IL-10.

### 2.6. Vitamin D and IBD

The health benefits of vitamin D are pleiotropic and include bone health, modulation of the immune system, antimicrobial protection, and mucosal integrity [46]. Deficiency of vitamin D is common in patients affected by IBD, in particular CD, due to malabsorption, and is worsened by reduced sunlight exposure and steroid treatment [47]. 

The positive effects of vitamin D are related to the improvement of epithelial barrier function [48] T-cells development, production of anti-inflammatory cytokines, and modulation on both innate and adaptive immunity [49], but even to antimicrobial peptides secretion [50]. In in vivo experiments, inadequate dietary intake of vitamin D promotes colitis development [51]. One possible explanation for this observation is that vitamin D deficiency produces intestinal dysbiosis, with a reduction of bacteria with anti-inflammatory properties (e.g., *Firmicutes*) and an increase in pathobiontic bacteria (e.g., *Bacteroides* and *Proteobacteria*) [52]. Moreover, even clinical observations have frequently found a link between low levels of vitamin D and a more aggressive course of IBD [53,54,55].

### 2.7. Emulsifiers and IBD

The western diet contains large amounts of emulsifiers, widely used in processed food to improve food appearance, texture, and palatability due to their intrinsic properties. Emulsifiers affect the gut microbiota, disrupt the mucosal barrier, and promote inflammation; in mice models, they induce metabolic syndrome, colitis, and translocation of *Escherichia coli* [56,57]. 

## 3. Nutritional Suggestions in Outpatient Management

Prevalence of malnutrition in IBD patients has been reported between 20% and 85%, with the highest prevalence occurring in hospitalized CD patients [58,59] and include both macro and micronutrients; the nutritional assessment is a crucial part of clinical evaluation [60]. Lean mass loss or sarcopenia should be regarded as a separate aspect of malnutrition and independently treated, even in patients with a normal body mass index (BMI) [61].

Several screening tools have been developed for the diagnosis of malnutrition, which includes phenotypic (age, weight loss, BMI) and etiologic (disease activity, food intake) criteria. The most important screening tools are the Saskatchewan IBD Nutrition Risk Tool, Malnutrition Inflammatory Risk Tool, Malnutrition Screening Tool, Nutrition Risk Screening (NRS) tool, and the Malnutrition Universal Screening Tool (MUST) [62]. 

A multidisciplinary approach and close collaboration with expert dieticians are crucial for the correct management of these patients, particularly those affected by short bowel syndrome and intestinal failure. 

About 15–40% of adults with IBD are obese, and an additional 20–40% are overweight, equally distributed between CD and UC [63]. Data suggests that obesity could promote rapid clearance of biologic drugs reducing the response to therapy and increases perioperative complications [63]. Obesity and sarcopenia in IBD patients can lead to sarcopenic obesity, which negatively impacts the patient’s status, increasing disability, mobility [61,63].

**Figure 1:**
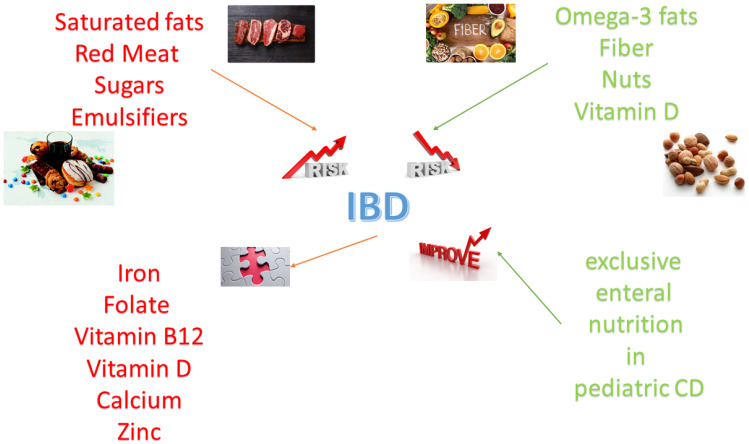
Pro-inflammatory and anti-inflammatory nutrients influence the risk of inflammatory bowel disease (IBD). Micronutrient deficiency is an important consequence of inflammatory bowel disease. Dietetic interventions, in particular exclusive enteral nutrition in children with Crohn’s disease, are effective in inducing remission.

A summary of the role of nutrients in inflammatory bowel disease is shown in Figure 1.

### 3.1. Micronutrients

In Table 1, the main micronutrients that deserve attention in patients affected by IBD are reported. 

#### 3.1.1. Iron

Iron deficiency is highly prevalent in patients affected by IBD, and about 20% of them suffer from iron deficiency anemia [70]. The main causes of iron deficiency are iron malabsorption in CD and loss from the gastrointestinal tract, especially in the case of active UC (whose hallmark symptom is bloody diarrhea). However, ulcers of colonic/ileal CD can lead to a less clinically evident, but not less severe, anemia [64]. A thorough assessment of iron metabolism parameters, including iron serum levels, transferrin (with transferrin saturation), and ferritin allows differentiating iron deficiency from anemia related to chronic diseases or combined anemia, which commonly occur. In iron deficiency anemia, low serum levels of iron, transferrin saturation, and ferritin are observed; conversely, in inflammatory anemia, ferroportin-1 is inhibited due to high hepcidin levels, and iron is sequestered in its deposits which leads to high ferritin and low transferrin serum levels [71]. In patients affected by CD with extensive small bowel inflammatory involvement or resection, iron malabsorption represents an additional causative factor. In combined anemia, ferritin levels may appear falsely normal, reflecting intermediate values between those induced by iron deficiency and inflammation, respectively [72]. However, even additional factors (renal failure, hemolysis) can contribute to worsening the anemia in IBD patients.

Iron is found in a lot of foods, including beef, liver, fish, poultry, eggs, legumes. Regarding the treatment of iron deficiency anemia, there is a heated debate regarding the best method of administration. Most authors favor intravenous (i.v.) administration due to a theoretical risk of IBD relapse due to oral iron administration [73], but recent publications have demonstrated the safety and efficacy of new oral iron formulations [74,75]. In our opinion, i.v. administration should be limited to patients with an iron deficiency anemia not responding or intolerant to oral iron administration. 

#### 3.1.2. Folate

Folate is a cofactor in DNA synthesis. Folate deficiency is the second most common micronutrient deficiency in patients with IBD, affecting about 30% of patients with CD and 10% of patients with UC [65]. The major causes are malabsorption (mainly in extensive CD with proximal small bowel malabsorption involvement or short bowel syndrome) or low intake, insufficient to counterbalance the blood loss from the gastrointestinal tract [76]. Further causes of folate deficiency are medications used to treat IBD like methotrexate or salazopyrine, which inhibit folate absorption [77]. In IBD patients with folate deficiency, celiac disease should be excluded by assessment of serum anti-tissue transglutaminase antibodies [78].

The main consequence of folate deficiency is megaloblastic anemia. Low folate levels can be associated with hyperhomocysteinemia, with an increased risk of thrombotic events [79]. 

Foods rich in folate are lentils, beans, vegetables, leafy greens, and citrus fruits. In addition to dietary advice, in the case of folate deficiency, 5 mg of folate per day is recommended with secondary use of methotrexate.

#### 3.1.3. Vitamin B12 (Cyanocobalamin)

Approximately 20% of CD patients develop vitamin B12 deficiency [66], because vitamin B12 is selectively absorbed in the terminal ileum, the most commonly affected segment in CD, which sometimes is even resected [80]. Although mucosal inflammation and/or insufficient oral intake are the major cause of deficiency in these patients, in case of severe or not fully explained deficiency, autoimmune gastritis should be excluded by measuring anti-parietal cell antibodies.

Clinical manifestations of vitamin B12 deficiency include megaloblastic anemia and peripheral neuropathy [81].

Foods rich in vitamin B12 are animal products, including fish, tuna, shellfish, beef, liver, poultry, eggs, dairy products. Vitamin B12 deficiency is supplemented with 5000 µg of intramuscular cyanocobalamin once monthly, and more recently, sublingual formulations have been introduced [82].

#### 3.1.4. Vitamin D

We treated the role of vitamin D in IBD pathogenesis and recurrence in the section dedicated to “*Nutrition in IBD—Pathogenesis*”.

Vitamin D deficiency is highly prevalent in IBD, involving up to 90% of malnourished and roughly 80% of apparently well-nourished patients [7,67]; a negative association was described between vitamin D serum levels and BMI [83].

Dietary intake of foods rich in vitamin D, such as dairy products (milk, yogurt), eggs, liver, cod liver oil, salmon, provide only a minor proportion of daily needs. Skin exposure to solar ultraviolet B radiation is the major source of vitamin D; in young adults, a summer sun exposure of about 25% of body surface (face and arms) for 15 min twice or thrice a week is equivalent to oral intake of 25 µg (1000 IU) [84]. All patients treated with steroids must be supplemented with at least 25.000 IU of cholecalciferol per month orally; patients with serum 25-OH vitamin D lower than 20 ng/mL must be supplemented with at least 25.000 to 50.000 IU of cholecalciferol per month orally [67]. If levels are <10 ng/mL, 50.000 IU per week for 6–8 weeks, then 800 IU daily are recommended [85]. In case of severe malabsorption, injectable formulations may be advisable, and the dosage of 300.000 IU intramuscularly every six months has been suggested [86,87].

#### 3.1.5. Zinc

Zinc is an essential trace element and plays an important role in wound repair, as well as being involved in the regulation of the immune system and in maintenance of balanced intestinal permeability [88]. Although the assessment of its serum levels is not the current practice of gastroenterologists, with the exception of patients with intestinal failure, it has been estimated that 15% of IBD patients suffer from zinc deficiency [69]. However, the reliability of the zinc measurement is questionable due to fluctuations with intake [68,89]. As low zinc levels have been associated with poor clinical outcomes in IBD, close monitoring and replacement of zinc in IBD patients should be suggested, especially in those with diarrhea [90].

Foods rich in zinc are meat (especially liver), seafood, eggs. Zinc supplementation requires 40–110 mg three times a day for 8 weeks orally and subsequent re-evaluation [91].

### 3.2. Macronutrients

Macronutrients include carbohydrates, proteins, and fats. In the section “*Nutrition in IBD—Pathogenesis*” we have already described how the western diet, rich in sugar, red meat, and saturated fats, and low in fiber, is considered pro-inflammatory [92] and could increase the risk of IBD. On the other hand, macronutrient deficiency may occur in patients with active IBD who reduce the oral food intake due to nausea/vomiting, abdominal pain, or fear of worsening symptoms during flares, as well as in case of stricturing CD. In case of severe macronutrient deficiency, such as in short bowel syndrome complicated by intestinal failure, a multidisciplinary approach with an expert dietician is mandatory and parenteral nutrition (PN) may be needed [93].

No specific dietary intervention has been demonstrated to promote remission in adult patients with active IBD [94]. The role of exclusive enteral nutrition (EEN), which in children with CD seems as effective as steroids in inducing remission [95], will be discussed in section “*Nutritional therapy during hospitalization*”. However, enteral nutrition support based on a polymeric liquid formula rich in TGF-β (Modulen IBD^®^) has proved to be effective in pediatric IBD [96]. Several “anti-IBD” diets have been proposed (e.g., the Specific Carbohydrate Diet, the Paleolithic diet, the IGG4 exclusion diet, and Semivegetarian diet), but currently, their diffusion among the majority of gastroenterologists and the data supporting their efficacy are scarce [97].

### 3.3. Sarcopenia

The assessment of sarcopenia in patients with active IBD was recently introduced into clinical practice. Sarcopenia, traditionally related to aging [98], is defined as a decrease of lean (skeletal) muscle mass with consequent decreased muscular strength, and it is a consequence of decreased mobility, chronic inflammation, malnutrition [99]. Patients with reduced oral intake, severe inflammation, or short bowel syndrome in CD, especially in the elderly, are prone to catabolism of muscle tissue. Drugs used to treat IBD, in particular systemic steroids, contribute to decrease muscle mass due to stimulating myostatin [100]. Sarcopenia represents a predictive factor for surgery and increases the rate of postoperative complications [101,102]. A mere evaluation of BMI in patients affected by IBD is misleading: In fact, body composition could be altered, with a reduced lean mass compensated by an increase in fat mass [103]. So, tests like hand-grip strength are mandatory to look for the disease [104]. Recently, it has been recently shown that the ratio between thyroid hormones, expression of decreased lean muscle mass, could be used as an indirect therapeutic biomarker of response to biological therapy in elderly patients with IBD [105]. Indeed, patients with an oral caloric intake insufficient to cover the metabolic needs (short bowel syndrome in CD, reduced oral intake due to obstructive symptoms, or severe inflammation), especially in the elderly, are prone to catabolism of muscle tissue. Physical activity is the most efficient way to increase muscle mass [106].

### 3.4. Obesity and IBD

The obesity “pandemic” of western countries do not spare patients affected by IBD: About 25% of these patients are obese and about 30% are overweight [107]. 

Obesity makes surgical resection more difficult [63,108], and increases the risk of anal and perianal fistulas, as well as perioperative complications. Obesity and sarcopenia act synergistically in determining physical impairment and metabolic disorders, mobility, and mortality [61]. In addition, obesity seems to reduce the response to biologic therapy, especially the fixed-dosing drugs, by increasing their clearance, which makes weight loss an adjunctive therapy in obese IBD patients [109,110]. 

Since dietary and lifestyle modification often obtains only short-term results, in selected cases of severely obese patients, bariatric surgery has been proposed as safe and effective alternative [111]. 

### 3.5. Clinical Trials

While a large body of literature was published on new drugs for IBD in recent years, few data are available for nutritional clinical trials.

Low fermentable oligosaccharides, disaccharides, monosaccharides, and polyols (FODMAP) diet is probably the most popular diet in functional gastrointestinal disorders, where it appears to be effective even in the long-term [112]. Some authors investigated its role even in the IBD setting. Bodini et al. [113] showed that six weeks of treatment with a low-FODMAP diet were able to reduce fecal calprotectin levels and to increase the quality of life of patients with IBD, probably related to the efficacy of low-FODMAP diet in reducing functional symptoms (like bloating, flatulence, or abdominal pain). In accordance, it is noteworthy that a randomized, double-blind, placebo-controlled, cross-over, and re-challenge trial conducted by Cox et al. [114] showed that fructans were able to exacerbate functional gastrointestinal symptoms in patients with IBD. These findings were confirmed in a subsequent, larger study aimed at evaluating the impact of low-FODMAP diet on symptoms, fecal microbiome and other markers of inflammation in IBD [115]: The low FODMAP diet reduced fecal abundance of microbes able to regulate the immune response, but had no significant effect on markers of inflammation; conversely, this diet induced significant improvements in functional symptom scores.

Another interesting clinical nutritional trial was conducted by Albenberg et al. [116], evaluating the impact of red meat consumption in patients with CD. Patients were divided in two groups according to the consumption of red meat (not more than 1 serving per month vs. a minimum of 2 servings per week) and followed for 49 weeks. Interestingly, the rates of CD relapses were similar in both groups.

## 4. Nutritional Therapy during Hospitalization

### 4.1. Hospitalization for Severe Disease

Nutritional interventions may improve outcomes for patients with IBD, especially in the most severe ones, and European Society for Clinical Nutrition and Metabolism (ESPEN) guidelines suggest screening for and manage undernutrition by using an appropriately trained multidisciplinary team [117]. Indeed, hospitalized patients with signs of malnutrition have higher risks of venous thromboembolism [118], non-elective surgery [119], longer hospital stay [119], and increased mortality [120]. Several indicators of malnutrition are currently accepted, but in clinical practice, the most used features are BMI < 18.5 kg/m^2^, unintentional weight loss exceeding 10% of total body weight and severe hypoalbuminaemia (<30 g/L) [121,122]. However, the American Society of Parenteral and Enteral Nutrition defines malnutrition as a status of any nutritional imbalance [123].

Initial findings of the importance of nutritional therapy were reported in the 1970s, when Voitk et al. showed an important clinical improvement of patients with severe CD treated with EEN [124,125]. EEN consists of either elemental, semi-elemental, or polymeric-based high-energy liquid formulas. An elemental diet is based on amino acids, sugars, fats, vitamins, and minerals, whereas non-elemental ones are composed of oligopeptide or whole-protein sources. Being amino acid-based, an elemental diet is completely absorbed in the duodenum and proximal jejunum, thus providing bowel rest for the distal small bowel and colon [126].

In children with active CD, EEN has been shown to be equally efficacious as steroids in order to induce clinical remission and mucosal healing, as well as to improve growth, correct micronutrient deficiencies, osteopenia, and anemia [127,128]. Therefore, it is suggested as a first-line therapy by ECCO/ESPGHAN guidelines [129]. The effect of EEN in inducing mucosal healing in IBD seems to be related primarily to modulation of gut microbiota [130,131], but some studies showed how it could be active in reducing pro-inflammatory cytokines [132,133].

On the other hand, in adult patients, enteral nutrition has lower effect and is less effective than corticosteroids in inducing remission [134,135]. However, the British Society of Gastroenterology suggests EEN for induction therapy in patients with mild-to-moderate CD who are interested in avoiding corticosteroids and are motivated to adhere strictly to EEN for up to 8 weeks [136]. Notably, a study by Heerasing et al. [137] in hospitalized patients with IBD showed that 25% of patients could avoid surgery due to EEN-induced remission. In Japan, EEN has been proposed as maintenance therapy in patients with CD, but its use in the USA and Europe is less widespread [138]. Interestingly, there is some evidence that the use of EEN in combination with infliximab could increase remission rates of response [139,140], and a meta-analysis showed that the combination of EEN with infliximab is more effective in maintaining clinical remission compared to infliximab alone (OR 2.7, CI 1.7–4.3) [141]. 

PN should be preferred in case of intestinal obstruction, intestinal ischemia, severe intestinal hemorrhage, high-output fistula, or ostomy, as well as in case of failure of enteral nutrition or inability to tolerate it [142,143]. Moreover, PN should be initiated even in case of severe malnutrition or short bowel syndrome due to previous bowel resections [144]. In the case of patients hospitalized for an acute inflammatory phase, particularly in the event of toxic megacolon and active fistulas, total parenteral nutrition (TPN) is the best option [145,146].

However, it is worth mentioning that PN, and especially long-term total parenteral nutrition (TPN), can be associated to infectious and thromboembolic complications (thrombophlebitis, deep venous thrombosis, and pulmonary embolism [147,148]). Patients with IBD are at risk for thromboembolism, which is related to multiple factors and includes an altered coagulation cascade, clinical factors, medications (steroids, tofacitinib), and surgery [149,150]. Notably, the risk of thromboembolism and infections related to PN in hospitalized patients is present even in pediatric ones [151]. Therefore, it is very important to start PN only in case of necessity.

Another important issue regarding hospitalized patients is fiber intake. We should report that a low-fiber diet is often ordered during hospitalization, but there are limited data in demonstrating its benefit. Nevertheless, the Academy of Nutrition and Dietetics and the American College of Gastroenterology recommends avoiding high-fiber foods in the presence of fistulas and strictures, as well as during CD flares [152,153]. The evidence about disease flares is more limited, although many patients note that high-fiber foods worsen IBD symptoms [154]. In the case of symptomatic strictures, it is logical to avoid high-fiber foods, which can induce a mechanical obstruction, though there are no data about the safe amount of fiber according to the extent of stricture [126].

### 4.2. Hospitalization for Surgery

Despite the use of biologic drugs, non-elective or elective IBD surgery rates have remained stable: Up to 70% of CD patients require abdominal surgery during their lifetime [155,156]. An optimal pre- and postoperative management can significantly improve the outcomes of patients who require surgery [157].

The most important problem related to nutrition in surgical patients is malnutrition. Indeed, the association between malnutrition and poor postoperative outcome was first reported in 1936, although not in IBD [158]. As expected, septic complications such as anastomotic leak, sepsis, and poor wound healing more frequently occur even in malnourished IBD patients [159]. A practical recommendation is to perform the Nutritional Risk Score (NRS) [160] and the Malnutrition Universal Screening Tool (MUST) [161] in all patients with IBD admitted to hospital for a surgical procedure. Patients with an NRS ≥ 3 have higher risks for complications after gastrointestinal surgery [162].

ECCO guidelines state that preoperative nutritional assessment should be performed for all patients with CD who need surgery [163]. Moreover, nutritional optimization prior to surgery, with enteral or parenteral nutrition, is recommended for those patients with nutritional deficiencies [163]. According to the ESPEN guidelines, the protocol for elective surgical patients includes avoidance of long-term fasting, integration of nutritional strategies into the overall management of the patient, metabolic homeostasis, and early mobilization [117]. Moreover, independently of the route of nutritional support, preoperative nutritional optimization in conjunction with stringent abdominal sepsis management and timely corticosteroid or immunosuppression withdrawal is able to avoid the need for a stoma in 92% of patients, with very few major complications and resulted in no postoperative mortality [164]. 

Although to correct nutritional imbalances is very important to increase postoperative outcome, no prospective randomized clinical trials have been conducted to assess the optimal route of nutritional supplementation before surgery, to the best of our knowledge. 

It is worth noting that a depletion of arginine from surgical stress can impair wound healing, but it can be overcome by a preoperative supplementation [165]. Treatment with perioperative and postoperative immune-modulating nutrients, such as arginine or omega-3 fatty acids has been recommended either as supplements or included in foods, particularly for malnourished patients undergoing gastrointestinal surgery in order to reduce infective complications and to reduce the length of stay [166].

TPN is able to improve body weight, nutritional status, disease activity scores, and inflammatory markers in a study of Jacobson et al. [167], and clinical remission was achieved in all patients on TPN before surgery. More interestingly, no postoperative complications occurred within 30 days in the TPN group, compared with 27.6% in the matched controls [167]. Another study showed that preoperative TPN was associated with a longer hospital stay, but also with a reduced small-bowel resection length [168]. Some studies showed that TPN was able to improve BMI but not to reduce surgical complications [169,170].

A meta-analysis of Brennan et al. [171] showed preoperative EN and PN reduced postoperative complications, but EN reduced postoperative morbidity and should be preferred as nutritional support, while PN should be reserved for those patients who are unable to tolerate EN. Indeed, the above-mentioned interesting study by Zerbib et al. [164] displayed that EEN was more effective in ameliorating nutritional parameters, such as serum albumin levels or, more importantly, in reducing the rate of intra-abdominal septic complications up to 3 months postoperatively. Accordingly, these results were confirmed in three Asian studies [172,173,174], which showed an effect of EEN in improving BMI, hemoglobin, albumin, and CRP levels, as well as in reducing the rates of infections and anastomotic leak. The lower rate of complications in patients treated preoperatively with EEN was previously demonstrated even by Smedh et al. [175], which compared a cohort treated with EEN with a retrospective cohort without this management, highlighting how the EEN-treated patients displayed lower surgical complications in the first 30 days. Furthermore, the study by Heerasing et al. [137] showed that activation of an EEN protocol avoided surgery in 25% of complicated CD patients.

For these reasons, ECCO guidelines suggest that a goal-driven PN should be considered only whenever EEN is difficult [163]. A summary of the study evaluating EEN and PN in surgical patients with IBD is displayed in Table 2.

It is worth mentioning the role of diet following surgery in IBD. Current guidelines suggest that patients should avoid fibers in the perioperative period [159]. However, a large survey conducted in North America showed that the avoidance of fiber is associated with a greater risk of CD flare in a 6-month period [43]. Therefore, we suggest the reduction of fiber intake only in the initial weeks following surgery, with a gradual reintroduction. 

In patients with ileal pouch after colectomy, the intake of antioxidants (such as cryptoxanthin, lycopene, vitamin A and vitamin C), mainly found in fruits, seem to reduce the rates of pouchitis [176,177]. This correlation could be explained by the modification in gut microbiota induced by the assumption of a higher quantity of antioxidants [177]. Accordingly, an adherence to the Mediterranean diet is associated with decreased fecal calprotectin levels after pouch surgery [178].

## 5. Conclusions

In the last decades, nutritional issues in IBD have received growing attention, focused on the pathogenic role of the western diet, as well as on the therapeutic impact of nutrition. Westernized dietary patterns have been recognized as risk factors for IBD because they promote a dysbiotic profile and intestinal inflammation [15].

In active disease, the accurate assessment of nutritional status, including sarcopenia and obesity, as well as the early treatment of protein/energy malnutrition and micronutrient/vitamin deficiencies, are strongly recommended [61]. Several nutritional strategies have been explored as exclusive (EEN for pediatric CD) or add-on therapy. PN is indicated only when enteral nutrition has failed or is contraindicated, particularly in hospitalized patients and in pre- and postoperative settings. The assessment and the treatment of malnutrition are crucial to improve the therapeutic outcome and prevent complications [117]. Therefore, the best therapy of IBD cannot be separated from an adequate nutritional management.

**Figure 2:**
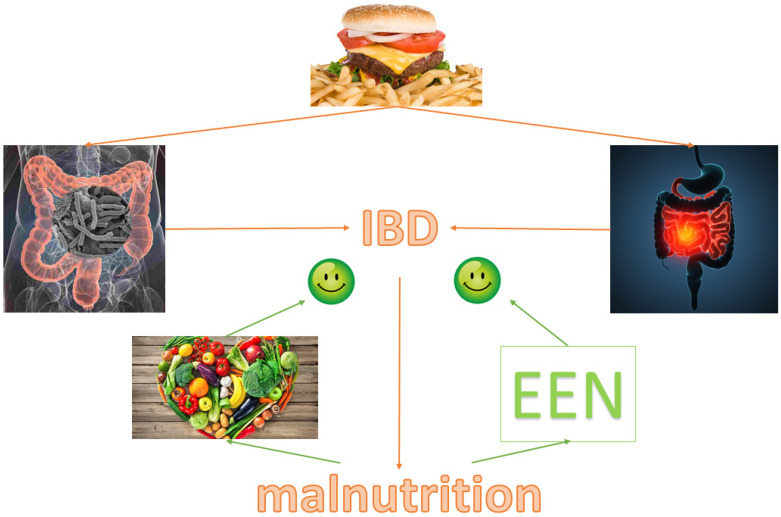
Western diet is a risk fact for inflammatory bowel disease because it causes intestinal dysbiosis and inflammation; one important consequence of inflammatory bowel disease is malnutrition. Micronutrient supplementation and dietary intervention could counterbalance these effects. IBD: Inflammatory bowel disease; EEN: Exclusive enteral nutrition.

Figure 2 displays our conclusions.

## Figures and Tables

**Table 1:** Micronutrients deficiency in patients affected by IBD.

Micronutrient	Population Target	Intervals	Supplementation	Consequences
Iron [64]	All patients	- Annually - if evidence of deficiency: Every 3 months for the first year, 6–12 months thereafter	- Mild or moderate anemia: - New oral formulation like ferrous bysglicinate chelate - Severe anemia or intolerant patients: - New intravenous formulation like ferric carboxymaltose	Anemia; Fatigue; Hair loss
Folate [65]	All patients	- Annually - if evidence of deficiency: Every 3 months for the first year, 6–12 months thereafter	5 mg of folate per day	Anemia; Fatigue; Hyperhomocysteinemia
Vitamin B12 [66]	All patients	- Annually - if evidence of deficiency: Every 3 months for the first year, 6–12 months thereafter	5000 microg intramuscularly or sublingual every month	Anemia; Fatigue; Hyperhomocysteinemia; Peripheral neuropathy
Vitamin D [67]	All patients	- Annually - if evidence of deficiency: Every 3 months for the first year, 6–12 months thereafter	- 25.000 to 50.000 UI of cholecalciferol per month orally - In case of severe malabsorption, 300.000 UI intramuscularly every six months	Osteoporosis; Worse disease course
Calcium [68]	Intestinal failure	- According to the deficiency	- Orally up to 3000 mg per day, plus intravenously according to the deficiency	Tetany; Osteoporosis
Zinc [69]	Intestinal failure	- According to the deficiency	- 40–110 mg three times a day for 8 weeks orally	Dysgeusia; Poor wound healing; Dermatitis; Failure to thrive

IBD, inflammatory bowel disease.

**Table 2:** The main studies evaluating nutritional support in surgical patients with IBD.

	Nutritional Route	Type of Study	Main Findings
Jacobson [167]	TPN	Case series (TPN vs. no nutritional support)	No complications in patients treated with TPN
Lashner et al. [168]	TPN	Retrospective study (TPN vs. no nutritional support)	Longer hospital stay in TPN; shorter resection length in TPN
Grivceva Stardelova et al. [169]	TPN	Retrospective study (TPN vs. no nutritional support)	Higher increase in BMI in TPN
Bellolio et al. [170]	TPN	Retrospective study (perforating vs. non-perforating disease)	TPN was associated with perforating disease
Brennan et al. [171]	TPN EEN	Meta-analysis TPN vs. EEN	EEN reduced postoperative morbidity
Zerbib et al. [164]	TPN EEN	Retrospective study evaluating complications in surgical patients	EEN reduced more than TPN septic complications
Li et al. [172]	EEN	Retrospective study EEN vs. no nutritional support	EEN reduced septic complications
Li et al. [173]	EEN	Retrospective study EEN vs. no nutritional support	EEN was associated with lower rate of stoma creation and with low postoperative complications
Wang et al. [174]	EEN	Prospective study EEN vs. no nutritional support	EEN increased nutritional parameters and was associated with low complications
Smedh et al. [175]	EEN	Prospective study on surgical techniques	Patients treated with EEN showed lower surgical complications

TPN: Total parenteral nutrition. EEN: Exclusive enteral nutrition.

## Data Availability

This paper is a review, and therefore do not have data to show.
